# Comparison of deep learning-assisted blinking analysis system and Lipiview interferometer in dry eye patients: a cross-sectional study

**DOI:** 10.1186/s40662-024-00373-6

**Published:** 2024-02-19

**Authors:** Yueping Ren, Han Wen, Furong Bai, Binge Huang, Zhenzhen Wang, Shuwen Zhang, Yaojia Pu, Zhenmin Le, Xianhui Gong, Lei Wang, Wei Chen, Qinxiang Zheng

**Affiliations:** 1 National Clinical Research Center for Ocular Diseases, Eye Hospital, Wenzhou Medical University, Wenzhou, 325027 Zhejiang China; 2 The School of Ophthalmology and Optometry, Wenzhou Medical University, 270 Xueyuan West Road, Wenzhou, 325027 Zhejiang People’s Republic of China

**Keywords:** Incomplete blinking, Deep learning model, Dry eye disease

## Abstract

**Background:**

Abnormal blinking pattern is associated with ocular surface diseases. However, blink is difficult to analyze due to the rapid movement of eyelids. Deep learning machine (DLM) has been proposed as an optional tool for blinking analysis, but its clinical practicability still needs to be proven. Therefore, the study aims to compare the DLM-assisted Keratograph 5M (K5M) as a novel method with the currently available Lipiview in the clinic and assess whether blinking parameters can be applied in the diagnosis of dry eye disease (DED).

**Methods:**

Thirty-five DED participants and 35 normal subjects were recruited in this cross-sectional study. DED questionnaire and ocular surface signs were evaluated. Blinking parameters including number of blinks, number of incomplete blinking (IB), and IB rate were collected from the blinking videos recorded by the K5M and Lipiview. Blinking parameters were individually collected from the DLM analyzed K5M videos and Lipiview generated results. The agreement and consistency of blinking parameters were compared between the two devices. The association of blinking parameters to DED symptoms and signs were evaluated via heatmap.

**Results:**

In total, 140 eyes of 70 participants were included in this study. Lipiview presented a higher number of IB and IB rate than those from DLM-assisted K5M (*P* ≤ 0.006). DLM-assisted K5M captured significant differences in number of blinks, number of IB and IB rate between DED and normal subjects (*P* ≤ 0.035). In all three parameters, DLM-assisted K5M also showed a better consistency in repeated measurements than Lipiview with higher intraclass correlation coefficients (number of blinks: 0.841 versus 0.665; number of IB: 0.750 versus 0.564; IB rate: 0.633 versus 0.589). More correlations between blinking parameters and DED symptoms and signs were found by DLM-assisted K5M. Moreover, the receiver operating characteristic analysis showed the number of IB from K5M exhibiting the highest area under curve of 0.773.

**Conclusions:**

DLM-assisted K5M is a useful tool to analyze blinking videos and detect abnormal blinking patterns, especially in distinguishing DED patients from normal subjects. Large sample investigations are therefore warranted to assess its clinical utility before implementation.

**Supplementary Information:**

The online version contains supplementary material available at 10.1186/s40662-024-00373-6.

## Background

Dry eye disease (DED) is one of the most common ocular diseases seen in the clinic. Previous studies reported the prevalence of DED, diagnosed with both symptoms and signs, to be 21%–55.7% in mainly middle-aged Asian adults [1–5]. However, it falls to 6.8%–34.4% in Caucasians at similar ages when DED is diagnosed subjectively in most studies [6–10]. It was proposed that Asians present poorer tear film stability, lipid layer quality and meibomian gland dropout than Caucasians [11]. Asians are also more likely to have a higher proportion of incomplete blinking (IB) [12], which is associated with pathological changes of meibomian glands and DED [13–16]. IB increases the exposure of ocular surface, prolongs the interblink interval, and thus leads to high tear osmolarity and tear film instability. IB could also disturb the meibomian lipid flow, making the tear film more vulnerable [16–19].

The number of IB and IB rate are the most common blinking parameters when evaluating the blinking patterns in DED [20–22]. It was reported that IB was related to ocular surface staining score and tear break-up time [22], suggesting the indication of DED diagnosis. Blinking is a complicated process since a variety of physiological and psychological factors are involved, so the recording of blinking has strict requirements on light source, light intensity, as well as the surrounding conditions. The analysis of blinking is not easy since minute movements can only be captured by highly-sensitive equipment. Currently, Lipiview (Johnson & Johnson Vision, Jacksonville, FL, USA) is an option for researchers to perform an objective evaluation of blinking patterns. Based on a 20-s video with a frame rate at 30 frames per second (FPS), it can generate a blinking profile and automatically calculate the number of blinks and IBs [22]. However, there are also some limitations that restrict its wide application: Lipiview uses flashing white light during the blinking examination, which may interfere with the spontaneous blinking activity [23]; moreover, the blinking video only lasts 20 s which may be not long enough to record the blinking pattern accurately since blinking activity fluctuates with time; lastly, it is unaffordable for most basic ophthalmic healthcare due to its high overhead cost.

There are studies using deep learning method to detect blinking completeness on full face images [24, 25]. Our recent publications also established a deep learning model (DLM) for blinking analysis on eye blinking images [26, 27]. It can analyze the blinking video recorded by Keratograph 5M (K5M; Oculus Optikgeräte GmbH, Wetzlar, Germany) which uses stable illumination and the recording time is not limited. In this study, we aimed to compare the DLM-assisted K5M system with Lipiview in detecting blinking parameters, as well as their practicality in the diagnosis of DED.

## Methods

### Participants

This cross-sectional observational study was approved by the institutional research ethics committee of the Eye Hospital of Wenzhou Medical University (2019-216-k-193) and adhered to the tenets of the Declaration of Helsinki. Written consent was obtained from all participants before examinations. A total of 35 DED patients and 35 age- and gender-matched normal controls (NC) were recruited at the Eye Hospital of Wenzhou Medical University. All participants were 18–35 years old to minimize the impact of age on blinking patterns [28]. The diagnostic criteria of DED were ocular surface disease index (OSDI) score ≥ 13 [29], and having one of the following signs: tear film break-up time ≤ 5 s or Schirmer’s I test (SIT) < 10 mm/5 mins, which was consistent with the Asia Dry Eye Society’s criteria [30]. Exclusion criteria included previous ocular trauma, ocular surgeries, active ocular diseases except for DED, eyelid diseases, wearing contact lens within one week before examinations, use of eye drops (except for preservative-free artificial tears more than 4 h before examination) and systemic medication, and pregnancy.

### Examinations

All examinations were conducted by two examiners (BF and BH), and a 5-min break were set between two tests. Participants were randomly assigned to one examiner who completed all tests after enrollment. The OSDI questionnaire was completed by interview to assess the symptomology first. Then, examinations were conducted in sequence: blinking assessments, tear meniscus height (TMH), non-invasive tear film break-up time (NIBUT), fluorescein tear film break-up time (FBUT), fluorescein corneal staining (FCS), SIT, and meibography.

Blinking assessments were conducted in the same room equipped with an adjustable light emitting diode, and illumination was fixed at 100 lux. Blinking assessments with Lipiview and K5M were performed on both eyes in a randomized order. K5M was set at “New picture/video” mode with conditions as Placido white 40, inner ring 50, fixation 20 in illumination, 0.5 magnification, high frame rate (30 FPS) and B/W (Black/White), which would provide a bright white light of around 300 lux. A 1-min blinking video was recorded for each eye.

**Fig. 1:**
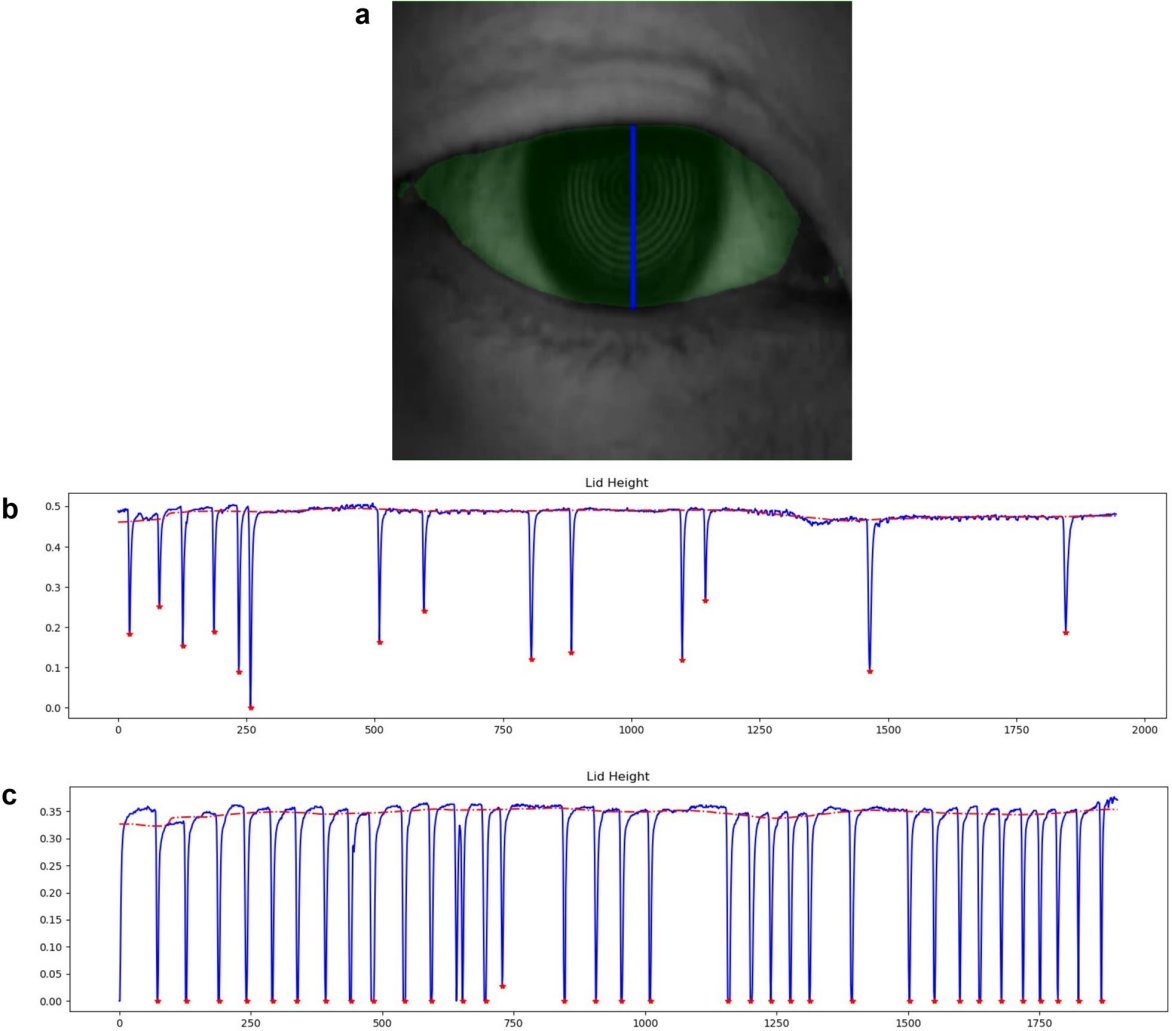
Deep learning model (DLM) analysis system of Keratograph 5M (K5M) recorded blinking video. **a** The interpalpebral zone is painted green according to the segmentation from DLM, and the vertical blue line indicates the maximum interpalpebral height (IPH) in this frame. The value of IPH in every frame is used to generate a blinking curve (**b, c**). **b** and **c** are the blinking curves from a normal control and a dry eye disease patient, respectively. The dotted red line represents the base value of IPH, and the red star marks each blinking

The previously established DLM for blinking analysis was introduced in the Additional file 1: Appendix S1 (Fig. 1). Before applying it in the current research, the segmentation performance of DLM were assessed on the new dataset consisting of 140 eyes. Blinking parameters generated from the DLM were also compared to the manually counted ones. The validation results are given in the Additional file 1: Appendix S1.

Lipiview uses a flashing white light which forms an interference pattern on tear film, to detect lipid layer thickness (LLT). At the same time, a built-in camera records a 30 FPS 20-s video of the tear film, and videos with a conformance factor over 0.7 were saved for blinking analysis [31]. The generated number of blinks and incomplete blinking were divided by 20 s (1/3 mins) for a frequency of per minute.

TMH and NIBUT were evaluated using K5M. An image was taken at 1.0× magnification under infrared light after two blinks, and TMH was measured on the lower tear meniscus below the pupil area. NIBUT was reported as the time period from the last blinking to the appearance of the first distortion, which was monitored on the grid reflection on tear film and averaged by three measurements (Supplementary video in Additional file 2).

FBUT and corneal staining were evaluated using silt-lamp microscope with 2% sodium fluorescein. FBUT was counted using a stopwatch, defined as the time taken after blinking twice for the first dry spot was observed on the tear film under Cobalt-blue light, and three measurements were averaged. FCS was assessed according to the Oxford Grading System under a yellow filter. Five areas of the cornea were graded individually from 0 to 5, with a total score ranging from 0 to 25 [32]. SIT was performed without anesthesia using a sterile strip with phenol red. The strip was placed in the outer one-third of the palpebral conjunctiva for 5 min with eyes closed. Meibography was evaluated at last by K5M. Images of both upper and lower meibomian glands were taken under infrared light, and the proportion of meibomian gland loss was graded according to Pult’s meiboscale [33].

### Statistics

Statistical analysis was performed with SPSS version 21.0 (SPSS, Inc., Chicago, IL, USA) and Prism 8.0 (GraphPad Software Inc, California, USA). Sample size was calculated with a significance level of 0.05 and power of 0.9. According to the difference of incomplete blinking rate in a previous study [26], a minimum of 28 participants was necessary for each group. Finally, 35 participants in each group were recruited. Kolmogorov-Smirnov test was used to determine the normality of data (*P* > 0.05). Bland-Altman analysis was performed to evaluate the consistency between the devices and measurements [34]. Blinking parameters from Lipiview were set as a gold standard, and the intra-individual biological variation (CV_I_) and inter-individual biological variation (CV_G_) were calculated. A minimum bias for Bland-Altman analysis can be derived as follows: ${\text{Minimum Bias }}\left( \%\right) = 0.375 \times \left( {{\text{CV}}_{{\text{I}}}^{2} + {\text{CV}}_{{\text{G}}}^{2} } \right)^{{0.5}}$. Intraclass correlation coefficient (ICC) of blinking parameters from the right and left eyes were calculated to evaluate the measurement consistency of Lipiview and DLM-assisted K5M on the basis that the two eyes from a single person have the same blinking pattern. Difference of evaluations between DED patients and NC were calculated using generalized estimating equations. The blinking parameters from different devices in groups were also compared using generalized estimating equations. Sex ratios between groups were compared using the χ^2^ test. Pearson’s or Spearman’s rank correlation was performed to assess the correlations between parameters. Receiver operating characteristic (ROC) curve was used to evaluate the diagnostic efficiency of blinking parameters, based on the diagnosis of each DED patient and the examination results of the right eye. A two-tailed *P* value less than 0.05 was considered as significant.

## Result

A total of 140 eyes of 70 participants (35 DED, 35 NC) were enrolled. The age and sex ratio showed no difference between groups (*P* ≥ 0.342). The DED group presented a higher OSDI score, shorter NIBUT and FBUT, lower SIT value, and higher FCS (*P* ≤ 0.023) compared with the NC group. However, no statistical differences were found in TMH, meiboscore, or LLT (*P* ≥ 0.123) between the two groups (Table 1).

**Table 1:** Demographic information and clinical assessments of participants

Parameter	DED (n = 70)	NC (n = 70)	*P* value
Age (years)	25 (23–25)	24 (23–24)	0.761
Gender (M:F)	4:31	8:27	0.342
OSDI score	29.35 ± 9.91	4.67 ± 3.86	< 0.001
NIBUT (s)	7.84 ± 4.31	14.97 ± 5.74	< 0.001
FBUT (s)	3 (2.75–4)	8 (6–10)	< 0.001
SIT (mm)	7 (4–14)	12 (5–25)	0.023
FCS	0 (0–1)	0 (0–0)	0.003

The data are presented as mean ± standard deviation, or median (interquartile range). Age, gender and OSDI score are compared between 35 DED subjects and 35 NC. NIBUT, FBUT, SIT and FCS are compared between 70 eyes from DED subjects and 70 eyes from NC

*DED* = dry eye disease; *n* = number of eyes; *NC* = normal controls; *M* = male; *F* = female; *OSDI* = ocular surface disease index; *NIBUT* = non-invasive tear break-up time; *FBUT* = fluorescein tear film break-up time; *SIT* = Schirmer’s I test; *FCS* = fluorescein corneal staining

**Fig. 2:**
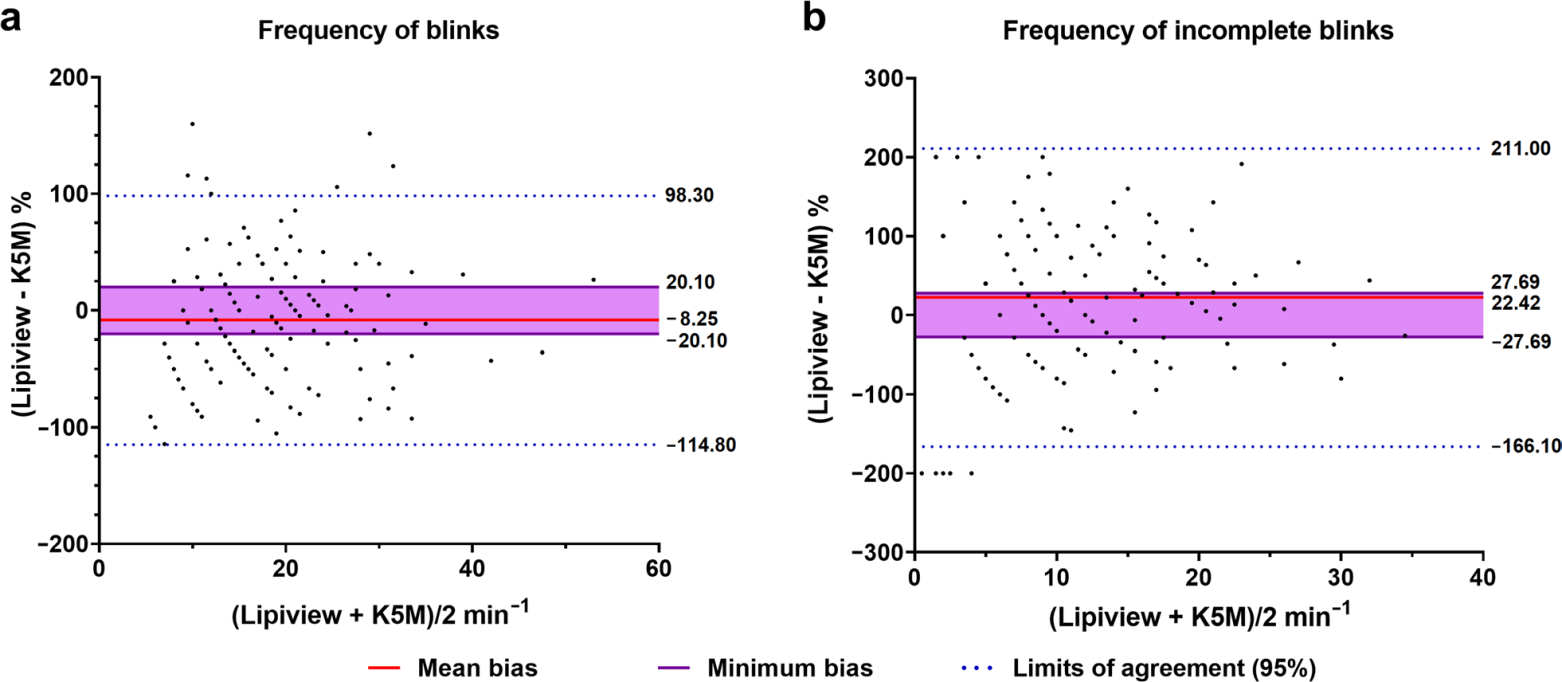
Bland-Altman plots showing the agreement in blinking parameters between devices. **a** The Bland-Altman analysis of frequency of blinks; **b** The Bland-Altman analysis of frequency of incomplete blinks. The limits of agreement were larger than the previous set minimum bias, and low consistency between devices were confirmed. K5M, Keratograph 5M

**Fig. 3:**
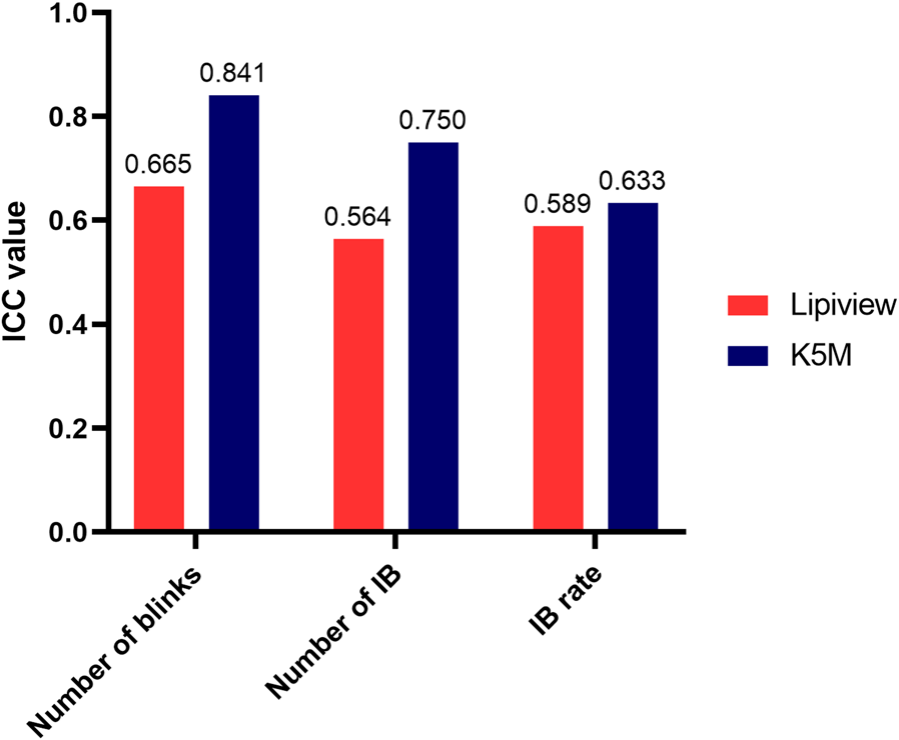
The consistency of measurement from K5M and Lipiview. The two eyes in one person have the same blinking pattern, and intraclass correlation coefficient (ICC) were calculated based on parameters from two eyes. K5M presented better consistency with higher ICC value in all measurements. IB, incomplete blinking; K5M, Keratograph 5M

Agreements between devices on measuring blinking parameters were evaluated using the Bland-Altman plot. The limits of agreements were wider than previously set minimum bias in both number of blinks and number of IB, and Lipiview captured a smaller number of blinks and higher number of IB than DLM-assisted K5M (Fig. 2). The consistency of measurements was also evaluated in both devices. The DLM-assisted K5M demonstrated higher ICC values in all three parameters (Fig. 3).

**Fig. 4:**
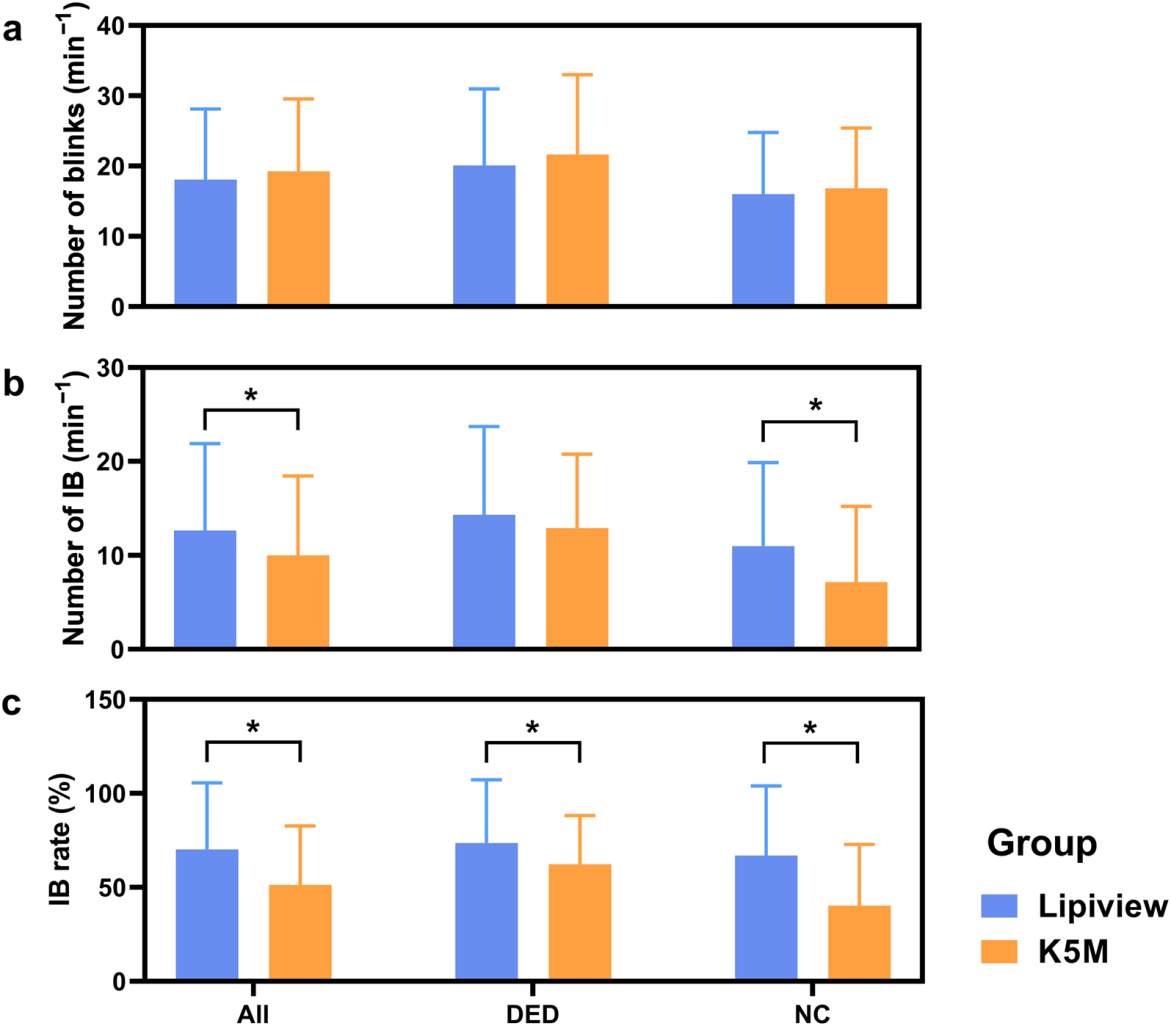
Comparison of blinking parameters between Lipiview and DLM-assisted K5M in the DED group, NC group and all participants. **a**, **b** and **c** showed the difference of number of blinks, number of IB and IB rate obtained from Lipiview and K5M in different groups, respectively. Lipiview captured higher number of IB and IB rate in all participants. Especially for the NC group, Lipiview exerts much greater values for the number of IB, and IB rate than DLM-assisted K5M. * indicates *P* < 0.05. DED, dry eye disease; NC, normal controls; DLM, deep learning model; K5M, Keratograph 5M; IB, incomplete blinking

Number of blinks, number of IB and IB rate were compared between both groups and devices using generalized estimating equations (Fig. 4). Interaction effects of devices and disease were significant when comparing IB rate between subgroups (*P* = 0.048). However, no interaction effects were found in comparison of number of blinks and number of IB (*P* ≥ 0.208). The main effect of device showed that videos from Lipiview gave a higher number of IB in all participants than that from DLM-assisted K5M (*P* = 0.006), as well as a higher IB rate (*P* < 0.001). The number of blinks from two devices were similar in both DED and NC subgroups (*P* ≥ 0.388). The number of IB from Lipiview were higher than that from DLM-assisted K5M in the NC group (*P* = 0.006), while it was not different in the DED group (*P* = 0.269). Besides, Lipiview demonstrated a higher IB rate in both the DED and NC groups, compared with the DLM-assisted K5M (*P* ≤ 0.047).

**Fig. 5:**
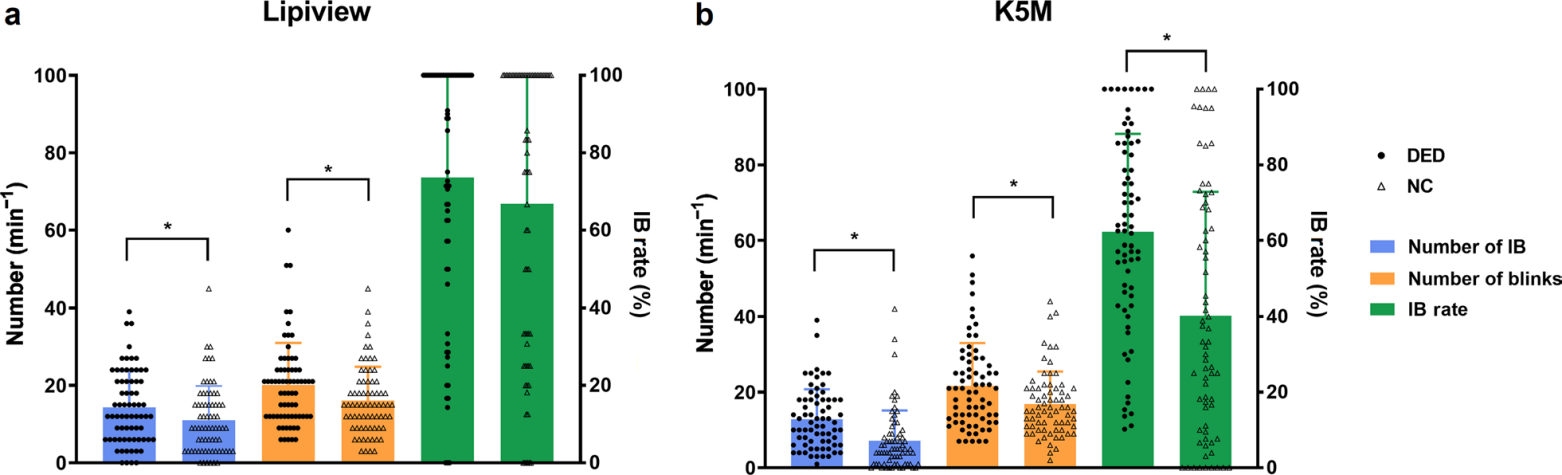
Comparison of blinking parameters between DED and NC with Lipiview (**a**) and DLM-assisted K5M (**b**). K5M revealed statistical differences in the number of blinks, number of IB and IB rate between the two groups, while Lipiview only showed the difference in the former two. * indicates *P* < 0.05. DED, dry eye disease; NC, normal controls; DLM, deep learning model; K5M, Keratograph 5M; IB, incomplete blinking

When comparing the ability to distinguish DED from normal subjects, DLM-assisted K5M captured a significantly higher number of blinks, number of IB, and IB rate in DED patients than those of NC (*P* ≥ 0.035). In contrast, Lipiview failed to show a difference in IB rate between the DED and NC groups (*P* ≥ 0.055; Fig. 5; Table 2).

**Table 2:** Comparison of blinking parameters from Lipiview and K5M between DED and NC

Device	Parameter	DED	NC	*P* value
Lipiview	Number of blinks (min^−1^)	21 (12–24)	15 (9–21)	0.055
	Number of IB (min^−1^)	12 (6–18)	9 (3–15)	0.083
	IB rate (%)	95.45 (50–100)	83.33 (29.33–100)	0.366
DLM-assisted K5M	Number of blinks (min^−1^)	20.5 (12.75–28.25)	15 (10.75–21)	0.035
	Number of IB (min^−1^)	11.5 (6–18)	5 (1–9)	0.001
	IB rate (%)	63.06 (44.81–85.71)	32.67 (9.89–69.06)	< 0.001

The data are presented in median (interquartile range)

*DED* = dry eye disease; *NC* = normal controls; *IB* = incomplete blinking; *DLM *= deep learning machine; *K5M* = Keratograph 5M

**Fig. 6:**
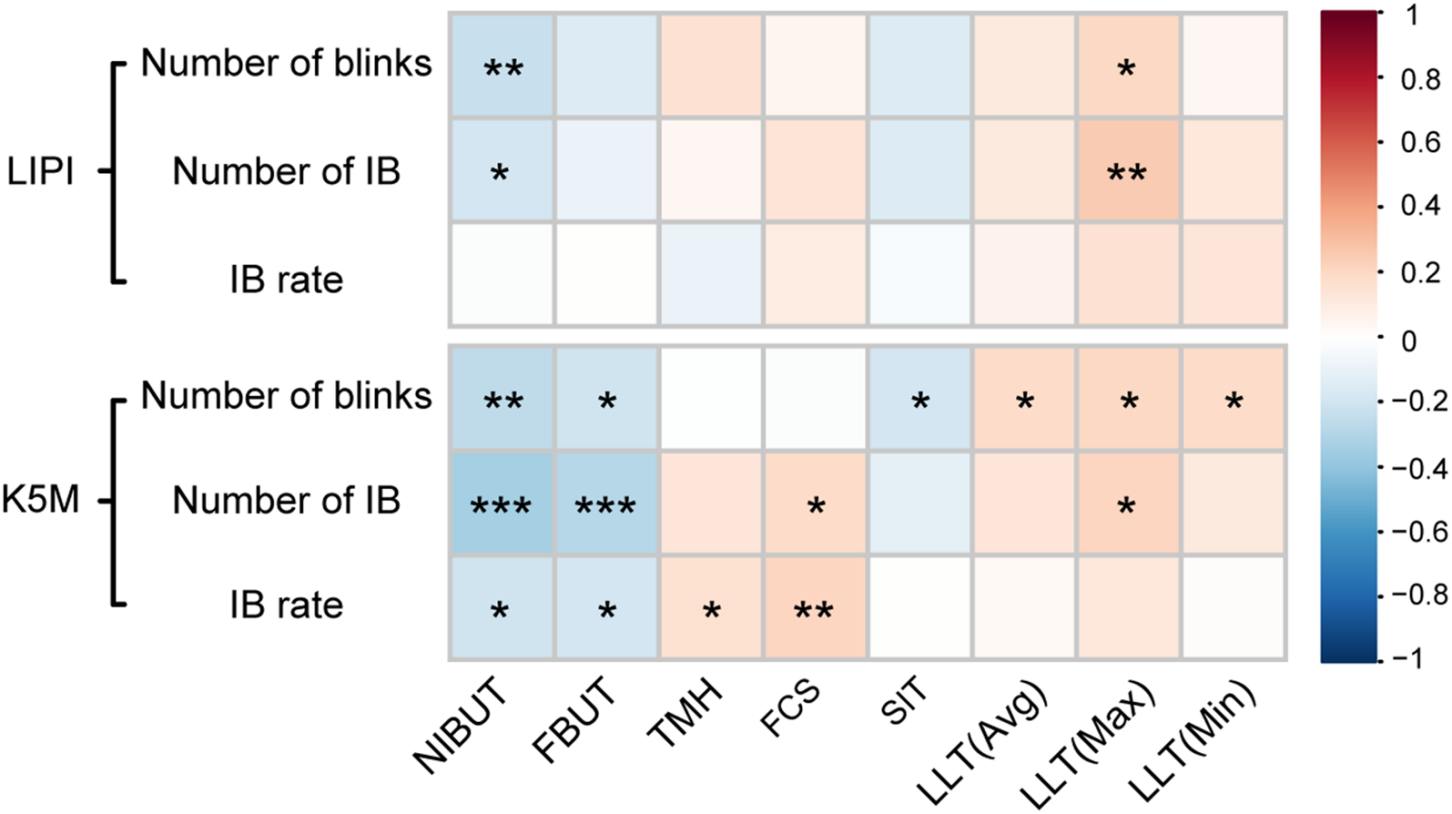
Heatmap of the correlation between clinical assessments and blinking parameters. Number of blinks, number of IB and IB rate were relative to NIBUT, FBUT, and other values. The stars in the table cells represent *P* values for the association test. * indicates *P* < 0.05, ** indicates *P* < 0.01 and *** indicates *P* < 0.001. IB, incomplete blinking; NIBUT, non-invasive tear film break-up time; FBUT, fluorescein tear film break-up time; TMH, tear meniscus height; FCS, fluorescein corneal staining; SIT, Schirmer’s I test; LLT, lipid layer thickness

Pearson’s correlation coefficient and Spearman’s rank correlation coefficient were calculated to evaluate the correlations between blinking parameters and DED measurements. The results from DLM-assisted K5M showed that the number of blinks was associated with NIBUT, FBUT, SIT, and LLT (R ≥ 0.180, *P* ≤ 0.033); and the number of IB and IB rate were relative to NIBUT, FBUT, FCS and other values (R ≥ 0.168, *P* ≤ 0.046; Fig. 6). However, the blinking parameters obtained from Lipiview showed fewer correlations with clinical evaluations; only number of blinks and number of IB were related to NIBUT and the maximum LLT (R ≥ 0.190, *P* ≤ 0.024).

**Fig. 7:**
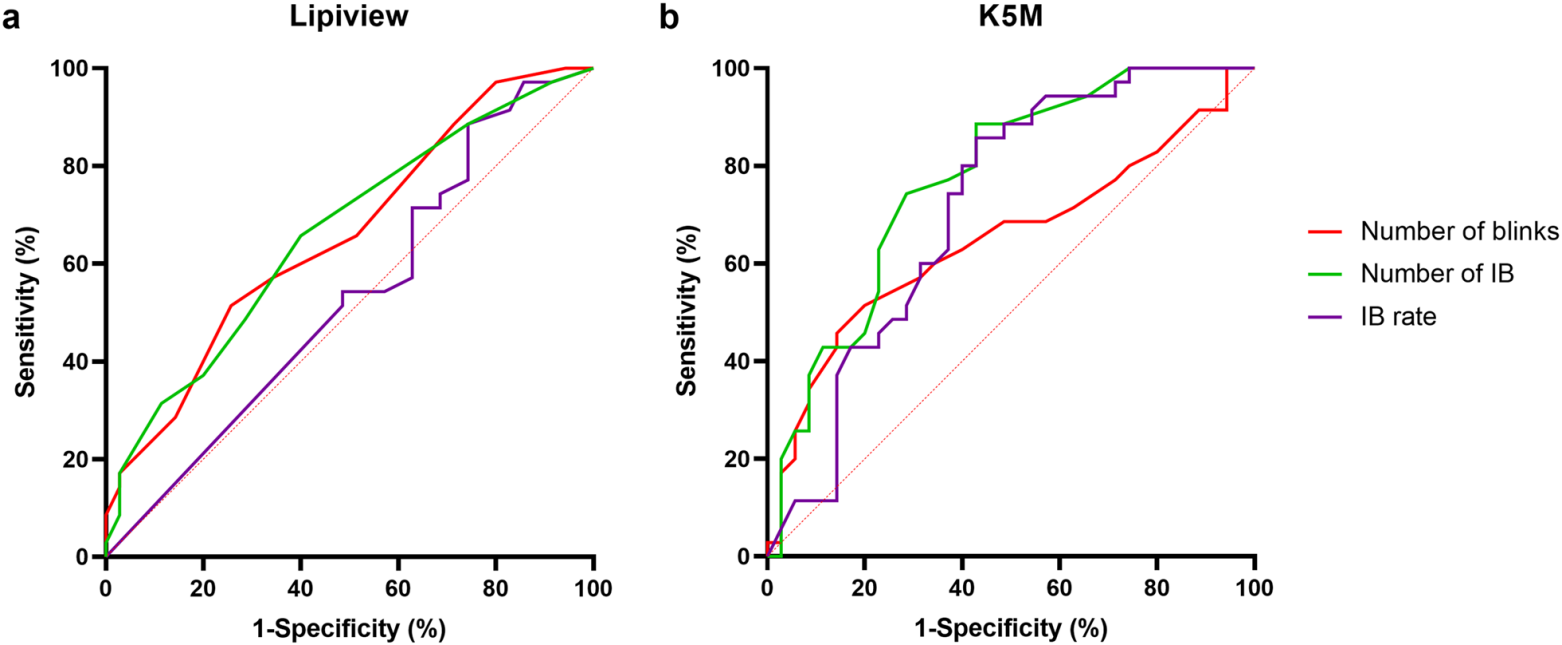
Receiver operating characteristic (ROC) curve based on parameters from Lipiview (**a**) and K5M (**b**). The parameters from K5M had higher sensitivity and specificity compared to Lipiview. No statistical difference was discovered between AUC. K5M, Keratograph 5M; IB, incomplete blinking; AUC, area under curve

Finally, ROC curves were generated from the right eyes of 35 DED patients to compare the efficiency of DED diagnosis between the two devices. Using the single diagnostic criterion of the number of IB from K5M with a cut-off point greater than eight yielded a sensitivity of 74.29% and specificity of 71.43%. Among all parameters between the two devices, the number of IB from K5M exhibited the highest area under curve (AUC) value of 0.773, which was not of significant differences nevertheless (*P* ≥ 0.18; Fig. 7; Table 3).

**Table 3:** Diagnostic efficiency of blinking parameters in DED from Lipiview and K5M

Parameter		Cut-off value	AUC	SE	*P* value	95% CI	Sensitivity (%)	Specificity (%)
Number of blinks	Lipiview	> 18	0.659	0.065	0.022	(0.532, 0.786)	51.43	74.29
	K5M	> 21	0.650	0.067	0.031	(0.518, 0.781)	51.43	80.00
Number of IB	Lipiview	> 9	0.662	0.065	0.020	(0.535, 0.789)	65.71	60.00
	K5M	> 8	0.773	0.056	< 0.001	(0.664, 0.883)	74.29	71.43
IB rate	Lipiview	> 0.25	0.531	0.070	0.651	(0.395, 0.668)	88.57	25.71
	K5M	> 0.389	0.716	0.063	0.002	(0.593, 0.839)	85.71	57.14

*DED* = dry eye disease; *AUC* = area under curve; *SE* = standard error; *CI* = confidence interval; *K5M* = Keratograph 5M; *IB* = incomplete blinking

## Discussion

Here, we found that blinking parameters generated from DLM-assisted K5M presented some differences *vs*. those from Lipiview with the same recording frame rate (30 FPS), which mainly lie in the higher sensitivity of DLM-assisted K5M to distinguish DED patients from normal subjects, and its more significant associations with clinical DED symptoms and signs.

Previous studies demonstrated that sagittal misalignment commonly exists in blinkings, which was termed “overblink”. Blinkings could be identified from an inferior-temporal view, and classified into incomplete ones, almost complete ones, and complete ones according to the degree of sagittal misalignment [35–38]. Lipiview detects blinks using a camera in the front view within 20 s, while K5M also records blinks in the front view but without limitation of time. The automatically classified blinks from Lipiview and DLM-classified blinks from K5M actually use the same definition of complete blinking containing both “almost complete blinks” and “complete blinks” [35]. However, Lipiview tends to confer a higher value of blinking compared with the DLM-assisted K5M. As a currently commercially available device for blinking analysis, Lipiview was reported to have the ability to provide blinking parameters related to diagnostic assessments of DED [39]. However, the flashing light it uses and the 20-s limit of recording time made the results vulnerable since the physiological process of spontaneous blinking could be violated. On the contrary, the K5M eliminates these limitations by using a stable moderate white illumination (300 lux) and unlimited recording time. Furthermore, we have established a DLM for accurate identification of blinking parameters for K5M-recorded videos in our recent reports [26, 40].

The current results showed that IB, as a potential biomarker for DED, were much higher in Lipiview than those obtained from the DLM-assisted K5M for the same subjects. The main reason may lie in the intense flashing light that Lipiview uses during blinking recording. It was previously reported that there is a functional relation between the visual cortex and the trigeminal nociceptive system, and flash light stimulation activated the visual cortex and triggered nociceptive blink reflex pronouncedly in healthy subjects [41]. Moreover, exposure to intense luminance could activate trigeminal nerve activity, increase parasympathetic outflow to the eye and cause ocular discomfort [42, 43]. Therefore, it is quite possible that the flashlight from Lipiview exerts an impact on the subjective comfort and interferes with the spontaneous blinking process. In fact, Lipiview was designed mainly for the quantitative measurement of LLT, which needs to use flashlight to form an interference effect on tear film. Thus, the blink analysis is not an advantage of it. However, the two devices in the current study both applied a certain testing condition, which might influence the spontaneous blinking process. Because the viewing distance, direction of light source and other settings of the devices were also different from the natural condition, these might have varied effects on blinking pattern. Accordingly, it is not confirmed yet in the current study whether Lipiview or DLM-assisted K5M can provide more accurate blinking parameters. Further investigations are needed to explore the impacts of illuminance, recording frame rate and recording time on blinking pattern for establishing a practical blinking analysis system. Novel DLM would also be developed to facilitate the blinking analysis system better in the near future.

It has been recognized in the past few years that DED patients have more IB than normal subjects at rest, and abnormal blinking pattern were related to the pathological change on the ocular surface [15, 44, 45]. Our current results demonstrated that the DLM-assisted K5M is more sensitive in distinguishing the abnormal blinking pattern from DED to normal subjects, which is of clinical value in the diagnosis of DED. Its consistency in acquiring blinking parameters was also better than Lipiview according to the uniformity of the parameters between the right and left eyes. Besides, it has been demonstrated that the spontaneous blinking activity presents a time-related reduction in 5 min [46]. A 20-s blinking video in Lipiview would cause more fluctuating results in blinking analysis, while K5M has an obvious advantage of unlimited recording time.

Our results also showed that the number of IB from DLM-assisted K5M, which showed the highest AUC although without statistical difference compared to other parameters, may help to diagnose DED. Nevertheless, the current sample size for ROC calculation may be inadequate, further validation from larger samples is needed. Increased IBs lead to unstable tear film and tear hyperosmolarity, which then aggravates DED progression [47, 48]. Tear hyperosmolarity induces ROS overgeneration, NLRP3 inflammation activation and inflammatory cytokines release [49–51], which is involved in the vicious circle of DED.

As a practicable management of DED, blink exercise has been proposed for years for abnormal blinking patterns in DED. Previous studies reported that the symptomology and signs of DED could be improved significantly after blinking exercise [52–54], but whether blinking exercise has a long-term effect, or how the blinking pattern was altered remains unknown. The current study may also help to understand the importance of blinking exercise and blinking pattern monitoring in DED patients. However, limitations also exist in the current study. The current DLM is trained with a dataset consisting of 1019 images, and it needs further improvement with larger dataset containing heterogeneous blinking images. Furthermore, the participants are mostly young adults, who may represent different blinking patterns from older DED patients. Additionally, the potential bias from the observers in DLM training and validation, and the unaccounted variables influencing the blinking process are somehow inevitable.

## Conclusion

We demonstrate here that the DLM-assisted K5M is a useful tool to analyze the blinking process and reveal abnormal blinking patterns. K5M provides a physiologically more comfortable recording condition than Lipiview, and the application of DLM made the analysis more objective and efficient, especially in distinguishing the DED patients from normal subjects. Therefore, the DLM-assisted K5M has the potential to analyze blinking for DED diagnosis; larger trials are warranted to determine its efficacy before implementation in routine practice.

### Supplementary Information

**Additional file 1: Fig. S1.** A series of extracted frames showing the movement of eyelids in blinking. **Fig. S2.** Examples of labeled images in infrared light dataset (left) and white light dataset (right). **Fig. S3.** Blinking profile generated by the deep learning model. The red line represents the base interpalpebral fissure height, and red stars mark the blinks in the video. The X-axis represents the video frames and Y-axis represents the ratio of IPH to the total height of the image (512 pixels). IPH, interpalpebral fissure height. **Table S1.** The performance of the trained DLM on the independent dataset in terms of the mean ± standard deviation of DSC, IOU, BAC, and SEN. **Table S2.** Comparison and consistency analysis of two blinking parameters obtained from extracted frames and DLM. **Table S3.** Comparison and consistency analysis of two blinking parameters obtained from extracted frames and slow-motion videos.

**Additional file 2: Video S1.** A generated video from the deep-learning assisted blink analysis system. The interpalpebral zone was labeled as green and the maximum interpalpebral fissure height was labeled as a vertical blue line according to the segemention result from deep learning model in every frame.

## Data Availability

The data used and analyzed in this study are available from the corresponding author upon reasonable request.

## Abbreviations

DLM: Deep learning machine
K5M: Keratograph 5M
DED: Dry eye disease
IB: Incomplete blinking
FPS: Frames per second
NC: Normal controls
OSDI: Ocular surface disease index
SIT: Schirmer’s I test
TMH: Tear meniscus height
NIBUT: Non-invasive tear film break-up time
FBUT: Fluorescein tear film break-up time
FCS: Fluorescein corneal staining
LLT: Lipid layer thickness
ROC: Receiver operating characteristic
AUC: Area under curve
