# Supplementary material for: Comparison of deep learning-assisted blinking analysis system and Lipiview interferometer in dry eye patients: a cross-sectional study
Source: Eye Vis (Lond). 2024 Feb 19;11:7. doi: 10.1186/s40662-024-00373-6 (PMC10875838; doi:10.1186/s40662-024-00373-6)
Supplement: Supplementary file 1 — Additional file 1: Fig. S1. A series of extracted frames showing the movement of eyelids in blinking. Fig. S2. Examples of labeled images in infrared light dataset (left) and white light dataset (right). Fig. S3. Blinking profile generated by the deep learning model. The red line represents the base interpalpebral fissure height, and red stars mark the blinks in the video. The X-axis represents the video frames and Y-axis represents the ratio of IPH to the total height of the image (512 pixels). IPH, interpalpebral fissure height. Table S1. The performance of the trained DLM on the independent dataset in terms of the mean ± standard deviation of DSC, IOU, BAC, and SEN. Table S2. Comparison and consistency analysis of two blinking parameters obtained from extracted frames and DLM. Table S3. Comparison and consistency analysis of two blinking parameters obtained from extracted frames and slow-motion videos. [file 40662_2024_373_MOESM1_ESM.docx]

**Additional file appendix**

1. **Introduction of the deep learning system for blinking analysis**

Due to the rapid eyelid closing and opening, incomplete blinks has always been hard to capture to human eyes in a blinking video. Therefore, we have developed a deep learning system for blinking analysis. The deep leaning model (DLM) is a texture-aware neural network based on the U-Net (termed TAU-Net) [1]. The architecture of the TAU-Net can be referred in the previous publication, where the TAU-Net had showed its advantages in identifying blinking comparing to other models.

- 1. **The dataset**

In a previous study, we collected two different types of blinking videos under infrared light and white light from 50 dry eye disease (DED) patients and 50 normal controls [2]. In most cases, the appearances of palpebral fissures in DED are same with normal controls. We randomly picked out 10 DED patients and 10 normal controls to establish datasets for the DLM. We first extracted all frames of the blinking videos, and frames capturing the movement of eyelids in blinking were selected (Figure S1). From the selected frames, 1019 images under white light and 1200 images under infrared light were randomly chosen and sorted into two datasets. The images were representative of the procedure of blinking, and sufficient for blinking analysis. All collected images were manually annotated using ImageJ software (National Institutes of Health, Laboratory of Optical and Computational Instrumentation, University of Wisconsin-Madison, Madison, WI, USA) (Figure S2). Then, the initial and masked images were resized to 512 x 512 pixels using a nearest neighbor interpolation scheme. The datasets were randomly divided into three disjoint subsets at a ratio of 8:1:1 for training, validation, and testing purposes, respectively.


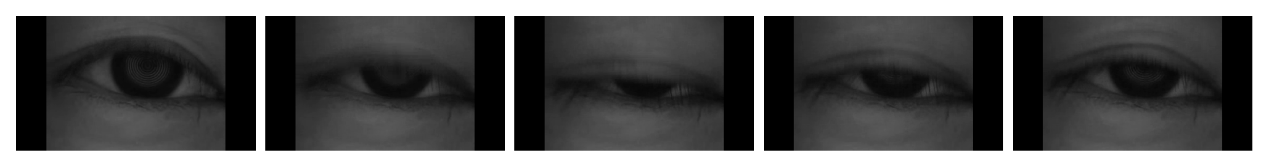


Fig. S1. A series of extracted frames showing the movement of eyelids in blinking.


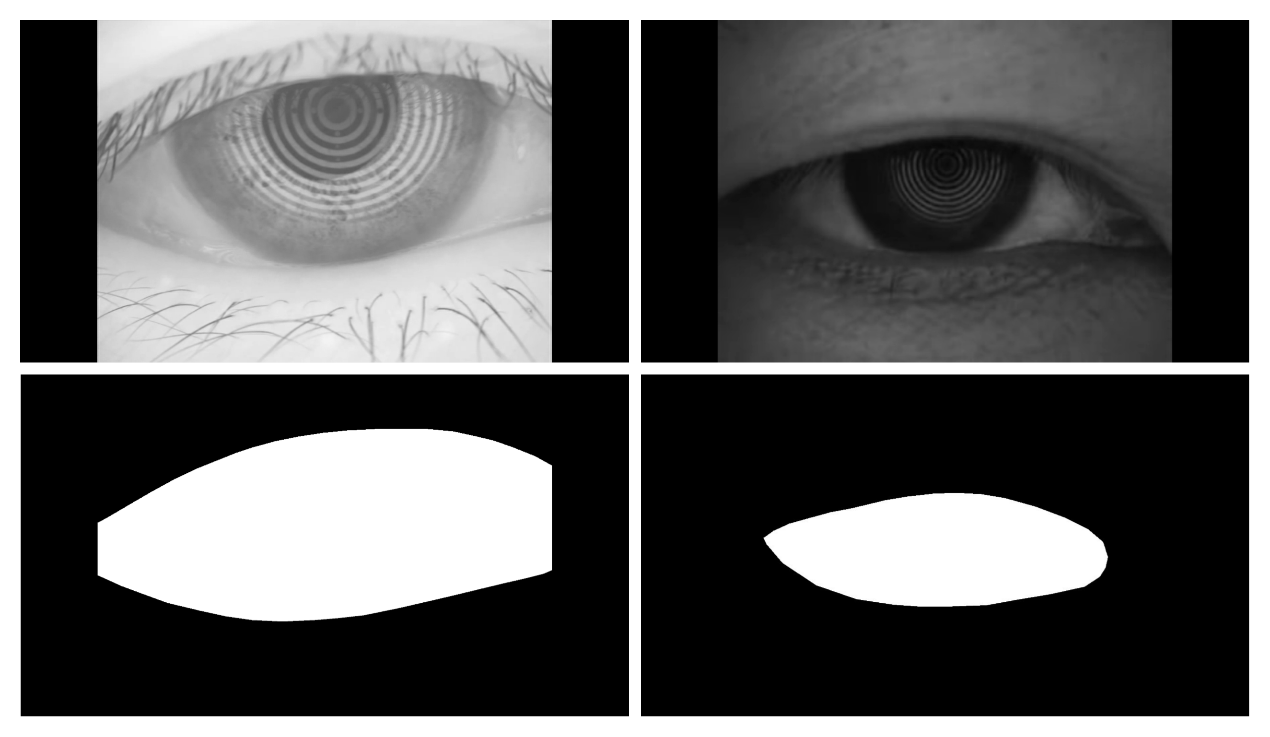


Fig. S2. Examples of labeled images in infrared light dataset (left) and white light dataset (right).

- 1. **Performance of the deep learning model**

For model assessment, a series of parameters including dice similarity coefficient (DSC), intersection over union (IOU), sensitivity (SEN), and balanced accuracy (BAC) were calculated as follows:

$$\begin{aligned} DSC=\frac{2Tp}{Tp+Fp+Tp+Fn}\#\left( 1 \right) \end{aligned}$$

$$\begin{aligned} IOU=\frac{Tp}{Tp+Fp+Fn}\#\left( 2 \right) \end{aligned}$$

$$\begin{aligned} ACC=\frac{Tp+Tn}{Tp+Tn+Fp+Fn}\#\left( 3 \right) \end{aligned}$$

$$\begin{aligned} SEN=\frac{Tp}{Tp+Fn} \#\left( 4 \right) \end{aligned}$$

$$\begin{aligned} BAC=1-\frac{Fn}{2(Tp+Fn)}-\frac{Fp}{2(Tn+Fp)}\#\left( 5 \right) \end{aligned}$$

Tp, Tn, Fp, and Fn denote true positive, true negative, false positive and false negative, respectively. These performance metrics range from 0 to 1, with a larger value denoting better performance.

The DSC, IOU, BAC and SEN of TAU-Net on white light image dataset were 0.9251±0.1671, 0.8868±0.1703, 0.9587±0.0819 and 0.9938±0.0063, respectively. The metrics on infrared light dataset were 0.9469±0.0604, 0.9046±0.0958, 0.9748±0.0185 and 0.9772±0.0294, respectively. All metrics were presented as mean ± standard deviation.

- 1. **Blinking analysis with the trained model**

A blinking analysis system was established based on the DLM. A blinking video shall be separated into single frames, and the palpebral fissure were segmented individually. The relative interpalpebral fissure height (IPH) was calculated according to the ratio of pixels. Based on the values of IPH in each frame, a profile can be generated to show the blinks in videos (Video S1, Figure S3). According to the ratio of the minimal IPH during one blink to the baseline IPH, blinks can be divided into complete and incomplete ones. Frequency of blinking and incomplete blinking, proportion of incomplete blinking, and average relative IPH can also be obtained from the blinking profile.


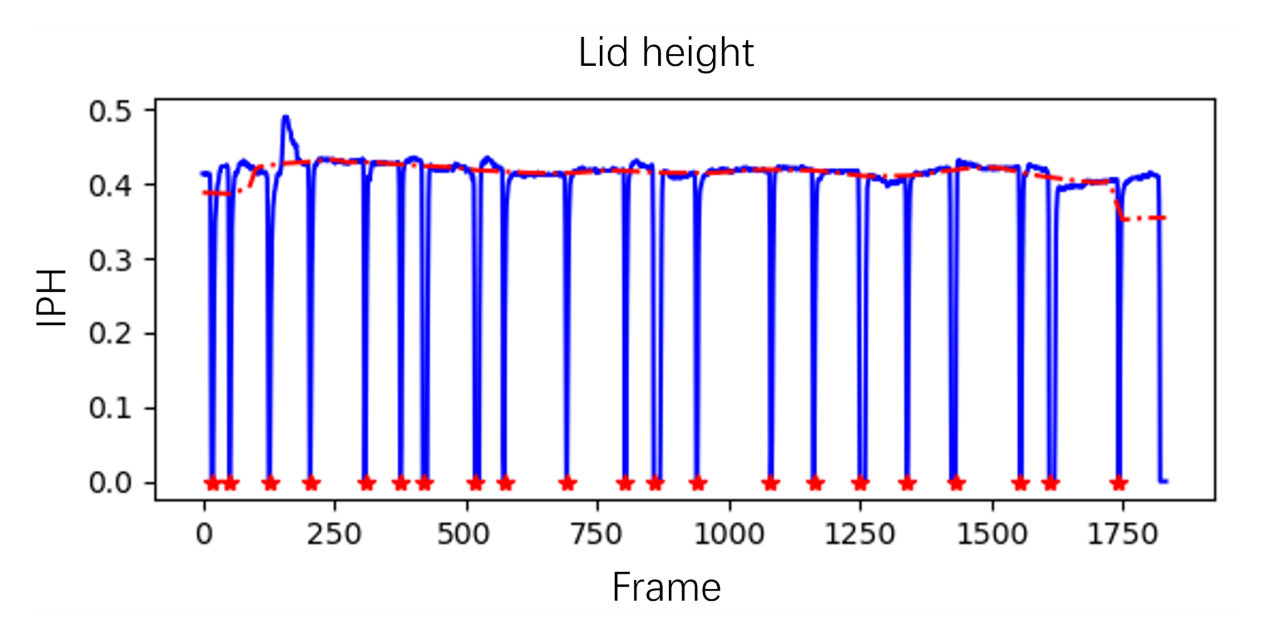


Fig. S3. Blinking profile generated by the deep learning model. The red line represents the base interpalpebral fissure height, and red stars mark the blinks in the video. The X-axis represents the video frames and Y-axis represents the ratio of IPH to the total height of the image (512 pixels). IPH: interpalpebral fissure height.

1. **Additional methods**
2. **Independent dataset for validation**

The current study recruited 35 DED patients and 35 normal controls for the comparison between two different blinking analysis system. A total of 140 one-minute blinking videos were recorded under white light on both eyes of the subjects. The appearance of palpebral fissure and movements of eyelid shall be diverse from the original dataset for training, which can be used to establish an independent dataset for validation. The first three blinks were selected in each video and the frames containing the whole blinking procedure were extracted. Among the extracted frames from one video, four frames were randomly picked to establish a new dataset named “New white light” with a total of 560 images. All images in the dataset were manually annotated using ImageJ software.

1. **Assessment of DLM performance**

By comparing the DLM segmented images with manually labeled ones, the performance of segmentation was assessed with metrics including DSC, IOU, BAC and SEN.

For assessing the performance on identifying blinking, frequency of blinks and incomplete blinks in 140 videos were calculated by a same investigator in different ways. The investigator firstly counted the blinking in all DLM-generated profiles without referring to videos. Then, the blinking videos were given in a random order. Investigator shall record the number of blinks and incomplete blinks when watching it played in slow motion at 0.5×. Finally, according to the frames extracted from previously checked blinking videos, investigator shall record the numbers as “golden standard”.

Paired t-test or Wilcoxon signed-rank test were used to compare the blinking parameters according to data normality. Intraclass correlation coefficient (ICC) were also calculated between groups.

1. **Additional results**
2. **Performance on segmentation**

Based on the manually labeled independent dataset containing 560 images from 140 eyes in different phase of blinking, the performance metrics of the current DLM were shown in Table S1.

Table S1. The performance of the trained DLM on the independent dataset in terms of the mean ± standard deviation of DSC, IOU, BAC, and SEN.

| Dataset | DSC | IOU | BAC | SEN |
| --- | --- | --- | --- | --- |
| New white light | 0.8910±0.1900 | 0.8348±0.1853 | 0.9518±0.0966 | 0.9279±0.1979 |

DLM = deep leaning model; DSC = dice similarity coefficient; IOU = intersection over union; BAC = balanced accuracy; SEN = sensitivity. The data is presented as mean ± standard deviation.

1. **Performance on blinking identification**

Frequency of blinking and incomplete blinking obtained from extracted frames were set as “golden standard”, and parameters from DLM-generated profiles and slow-motion videos were compared to it respectively. The parameters obtained from extracted frames and DLM were same in all videos (Table S2). However, frequency of incomplete blinking obtained from slow-motion videos were different from the “golden standard” (Table S3).

The parameters obtained from DLM-generated profile would be used in the current study now that it had been validated.

Table S2. Comparison and consistency analysis of two blinking parameters obtained from extracted frames and DLM.

| Blinking parameter | Extracted frames | DLM | ICC | *P* value |
| --- | --- | --- | --- | --- |
| Frequency of blinking (min^−1^) | 17 (12–25) | 17 (12–25) | 1 | 1 |
| Frequency of incomplete blinking (min^−1^) | 8 (4–14.75) | 8 (4–14.75) | 1 | 1 |

DLM = deep learning model; ICC = intraclass correlation coefficient. The data are presented as median (interquartile range).

Table S3. Comparison and consistency analysis of two blinking parameters obtained from extracted frames and slow-motion videos.

| Blinking parameter | Extracted frames | Slow-motion videos | ICC | *P* value |
| --- | --- | --- | --- | --- |
| Frequency of blinking (min^−1^) | 17 (12–25) | 17 (12–25) | 1 | 1 |
| Frequency of incomplete blinking (min^−1^) | 8 (4–14.75) | 7 (4–13.75) | 0.981 | <0.01 |

ICC = intraclass correlation coefficient. The data are presented as median (interquartile range).

**References for supplementary appendix**

1. Zheng Q, Zhang X, Zhang J, Bai F, Huang S, Pu J, et al. A texture-aware U-Net for identifying incomplete blinking from eye videography. Biomed Signal Process Control. 2022;75:103630..

2. Zheng Q, Wang L, Wen H, Ren Y, Huang S, Bai F, et al. Impact of incomplete blinking analyzed using a deep learning model with the Keratograph 5M in dry eye disease. Transl Vis Sci Technol. 2022;11(3):38.
